# Quality of Life and Persistence of Symptoms in Outpatients after Recovery from COVID-19

**DOI:** 10.3390/medicina58121795

**Published:** 2022-12-06

**Authors:** Lizeth Guadalupe Gutiérrez-Canales, Carolina Muñoz-Corona, Isaac Barrera-Chávez, Carlos Viloria-Álvarez, Alejandro E. Macías, Eduardo Guaní-Guerra

**Affiliations:** 1 Fellow of the General Directorate of Quality and Health Education, Ministry of Health, León 11410, Mexico; 2 Department of Medicine and Nutrition, University of Guanajuato, San Carlos La Roncha, León 37660, Mexico; 3 Department of Research, Hospital Regional de Alta Especialidad del Bajío (HRAEB), San Carlos La Roncha, León 37660, Mexico

**Keywords:** quality of life, COVID-19, SARS-CoV-2, SF-36 questionnaire, persistent COVID-19 symptoms, long COVID

## Abstract

*Background and Objectives*: Patients infected with SARS-CoV-2 can have persistent symptoms after acute illness, which affects their quality of life (QoL). Research and data about this topic in Latin American ambulatory patients are scarce. *Materials and Methods*: We conducted an observational, prospective, transversal, and analytical study. To measure QoL, we used a validated Spanish version of the MOS/RAND 36-Item Short Form Health Survey (SF-36). *Results*: We included 206 outpatients in the study. A total of 73.3% patients had persistence of one or more symptoms. The most frequent persistent symptoms were fatigue (36.9%), anxiety (26.2%), and headache (24.8%). No statistically significant difference in the SF-36 QoL scores and the frequency of persistent COVID-19 symptoms was found when comparing the ≤5 and >5 months groups, except for myalgia, which was less frequently observed in the >5 months group after COVID-19 (26.2% vs. 14.1%, *p* < 0.038). Female gender was associated with an increased risk of persistence of symptoms (OR = 2.95, 95% CI 1.56–5.57). Having comorbidities/sequelae attributed to COVID-19 and persistence of COVID-19 symptoms were associated risk factors for poor physical component summary (PCS); on the other hand, female gender, anxiety, and depression were associated with poor mental component summary (MCS). *Conclusion*: Most outpatients had persistent COVID-19 symptoms after infection. Persistence of symptoms was associated with poor MCS and PCS. It is important to follow-up not only patients discharged from the hospital after SARS-CoV-2 infection, but also those under ambulatory management to provide them with rehabilitation and psychological therapy to improve their QoL.

## 1. Introduction

Since the first case of COVID-19 was reported in December 2019, it has been demonstrated how the SARS-CoV-2 infection affects daily life and social and economic development as it became a pandemic that caused millions of deaths [1,2]. In people who develop clinical illness, the respiratory system is the most severely affected. The virus can non-surprisingly affect any organ in the body; the virus binds to angiotensin converting enzyme 2 (ACE2) receptors present in vascular endothelial cells, lungs, heart, brain, kidneys, intestine, liver, pharynx, and other tissues where it may lead to organ malfunction [3]. Injury to the organs may become apparent long after the acute infection has subsided, and chronic injury may occur [4].

Therefore, those infected with SARS-CoV-2 can have persistent symptoms for months after acute illness; this has been called long-COVID-19 or post-COVID-19 syndrome by some authors [5,6]. A high proportion of the patients report persistent symptoms such as fatigue, dyspnea, chest pain, smell dysfunction, and sleep disorders [7,8,9]. However, most reports are from patients who were hospitalized; for example, Evans et al. observed that only 28.8% of patients described themselves as fully recovered whereas 92.2% reported at least one persistent symptom after 5 months of infection [10]. Similarly, Carfi et al. described that a total of 87% patients had at least one or more persistent symptom and only 12.6% were completely free of any COVID-19 symptoms in a mean of 36.1 ± 12.6 days after discharge [5]. On the other hand, there are few studies on persistent symptoms in non-critical patients and although the percentage is variable (53% and 32.7%), these studies report that outpatients may also have persistent symptoms after COVID-19 infection [11,12]. Hence, it is important to evaluate the recovery of patients who had COVID-19, the consequences of the disease, and how their quality of life (QoL) was affected, so that timely interventions can be made [13]. To our knowledge, persistence of COVID-19 symptoms and QoL has not been evaluated in the Latin American population after SARS-CoV-2 infection with ambulatory management.

The present study aimed to identify sequelae and persistent symptoms, as well as their influence on QoL, in outpatients who had recovered from COVID-19. 

## 2. Materials and Methods

According to the STROBE guidelines [14], an observational, prospective, transversal, and analytical study was conducted between March and June 2021. A total of 206 ambulatory patients, including men and women older than 18 years, who had recovered from COVID-19 (2 to 12 months after the onset of symptoms) from different locations in the state of Guanajuato, Mexico, were included in the study. SARS-CoV-2 infection was confirmed by real-time reverse transcription–polymerase chain reaction (RT-PCR) during acute illness. Patients included in the study were unvaccinated against SARS-CoV-2 at the time of infection. Patients who completed less than 80% of the SF-36 questionnaire were not included nor were patients that required hospitalization. The study was approved by the Ethics and Research Committee of the Regional Hospital of High Specialty of the Bajío, state of Guanajuato, Mexico (approval number CEI-008-2022). Participants included in the study provided their informed consent.

Authors interviewed the patients after COVID-19 infection and allocated them into one of two groups as follows: patients who persisted with symptoms less than or equal to 5 months after COVID-19 infection constituted the first group, and those who persisted with symptoms after 5 months constituted the second group; this grouping was based on a previously report [15]. A prospective database with a standard questionnaire was used to obtain the following variables: epidemiological data, comorbidities, date at the onset of symptoms, persistence of symptoms (joint pain, fatigue, myalgia, dyspnea, headache, anxiety, depression, vertigo, cough, chest pain, sore throat, smell dysfunction, memory loss, insomnia/poor sleep quality, and diarrhea), complications, and sequelae attributed to COVID-19. Depression and anxiety were measured using a Spanish-validated version of the Patient Health Questionnaire 9-item depression scale (PHQ-9) and 7-item Generalized Anxiety Disorder scale (GAD-7). These tools have been identified as some of the most reliable screening tests to identify depression and anxiety. In a recent meta-analysis to assess the accuracy of PHQ-9 to screen for depression, the overall sensitivity was 0.88 (95% CI 0.83 to 0.92) and the specificity was 0.85 (95% CI 0.82 to 0.88) [16]; whereas for GAD-7, a systematic review identified a pooled sensitivity of 0.83 (95% CI 0.71–0.91) and specificity of 0.84 (95% CI 0.70–0.92) for anxiety [17].

The MOS/RAND SF-36 questionnaire in Spanish, validated for the Mexican population, was used to measure QoL [16]. The SF-36 measurement characteristics have been studied extensively to assess its reliability and validity [15,18,19]. The questionnaire consists of 36 items aimed at measuring the following eight dimensions: physical functioning (PF, limitations in performing physical activities such as bathing or dressing), role-physical (RP, limitations in work and other daily activities as a consequence of physical health), bodily pain (BP, how severe and limiting pain is), general health (GH, how general personal health is perceived by the patient), vitality (VT, feeling tired and worn out vs. feeling energetic), social functioning (SF, interference with regular social activities because of physical or emotional problems), role-emotional (RE, limitations in work and other daily activities as a consequence of emotional problems), and mental health (*MH*, feeling nervous and depressed vs. peaceful, happy, and calm). Further, the score health change (HC, change in general health status from the previous year) was included. Scores for each item ranged from 0 to 100, with higher scores indicating better health. Two additional scores were calculated with the oblique (correlated) method, which included the physical component summary (PCS) and the mental component summary (MCS). The PCS was derived from positive weights for the PF, RP, BP, GH, and VT scales and negative weights for the SF, RE, and MH scales. The scoring algorithm for MCS included positive weights for VT, SF, RE, and MH scales and negative weights for the PF, RP, BP, and GH scales. These scores were calculated following the methodology proposed by Ware and Hays, the creators of the scores [20,21,22,23]. The PCS and MCS are presented as T-scores [22].

Quantitative variables were described using the median with interquartile range (IQR) and mean ± standard deviation (SD). Categorical data were described using absolute and relative frequencies. The normal distribution of continuous variables was assessed using the Kolmogorov-Smirnov test. Regarding the comparative analysis among the two study groups, categorical data were analyzed using Chi-squared test and Fisher’s exact test, while the SF-36 scores among the different groups were compared using the Kruskal-Wallis test. Odds ratios were calculated for qualitative dichotomous variables. Statistical analysis was performed using VassarStats [24]. Univariable and multivariable logistic regression models were used to explore risk factors associated with poor health-related quality of life (HRQoL). All variables that were significant (*p* < 0.05) in the univariate logistic regression models were included in the multivariable logistic regression model performed by hierarchical selection. Poor HRQoL was defined as scores of PCS or MCS less than 50, as previously described in other studies [25,26]. The logistic regression analyses were performed with NCSS 12.0.2 Statistical Software (NCSS, LLC, Kaysville, UT, USA, 2018).

## 3. Results

The 206 outpatients included in the study were interviewed at a mean time of 163.6 ± 4.2 days after the onset of COVID-19 symptoms. The female-to-male ratio was 1.67:1. At the time of the survey, the median age was 28 (IQR, 22–45.5) years. Chronic associated diseases were present in 75 (36.4%) patients, the most frequent being overweight (14.1%), depression (9.2%), hypertension (7.3%), and diabetes (4.9%) (Table 1).

During the ambulatory management, 23 patients (11.2%) received home supplemental oxygen. Complications and sequelae attributed to COVID-19 were present in 16 (7.8%) patients, with inability to walk being the most frequent complication in 12 (5.8%) patients. Medication management included a macrolide antibiotic in 72 (35%) patients, both vitamin D and corticosteroids in 56 (27.2%) patients, and ivermectin in 47 (22.8%) patients.

**Figure 1:**
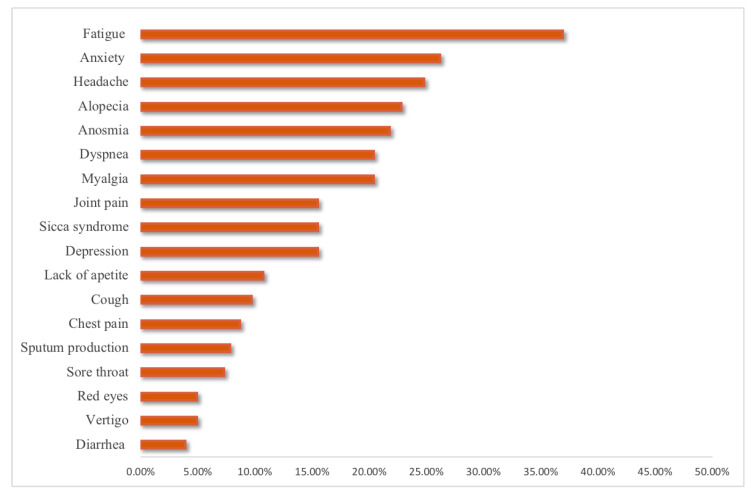
Persistence of symptoms in outpatients who had recovered from COVID-19.

Among the 206 study subjects, 151 (73.3%) had persistence of one or more symptoms, 83 (40.29%) had three or more symptoms, 42 (20.38%) patients reported two symptoms, and 27 (3.10%) reported only one persistent symptom, with fatigue (36.9%), anxiety (26.2%), headache (24.8%), and alopecia (22.8%) being the most frequent ones (Figure 1).

To assess the association influence of gender, age, comorbidities, and treatment on symptom persistence, a comparison was performed with asymptomatic patients. A higher proportion and increased risk of persistent symptoms were observed in female symptomatic patients when compared to female asymptomatic patients (69.5% vs. 43.6%, OR = 2.95, 95% CI 1.56–5.57, *p* = 0.001), or those treated with macrolides (41.1% vs. 18.2%, OR = 3.13, 95% CI 1.47–6.67, *p* = 0.003). Smoking was observed in higher proportion of the asymptomatic group (20.5% vs. 38.2%, OR = 0.42, 95% CI 0.21–0.82, *p* = 0.012) (Table 2). 

As mentioned before, to compare the persistence of symptoms and QoL in different periods of time, it was decided to separate the patients into two groups after COVID-19 infection; the first group suffered from persistent symptoms for less than or equal to 5 months, and the second group had persistent symptoms for more than 5 months. 

To determine whether the groups were similar enough to perform further statistical analysis, the general characteristics, gender, and comorbidities were compared, which identified no significant differences (Table 3).

Regarding complications and sequelae due to COVID-19, inability to walk, neuro/myopathy, and myocardial infarction/heart failure showed no significant difference between the two groups. On the other hand, when comparing persistent symptoms, a statistically significant difference was observed only for myalgia, which was more frequent in the ≤5 months group (26.2%) when compared to the >5 months group (14.1%) (*p* < 0.038) (Table 4). 

The SF-36 questionnaire analysis showed that for all study patients, the Health Change score (HC) was the most affected scale (median score of 50, IQR 25–75), followed by the Vitality domain (VT) (median score of 55, IQR 45–70) and Mental Health domain (MH) (median score 60, IQR 48–76). Characteristically, the oblique mental component summary (MCS) (median score 45.4) presented lower scores than the oblique physical component summary (PCS) (median scores 50.2) (Table 5).

When comparing the SF-36 scores between the two groups (symptomatic vs. asymptomatic), the most affected score was, once again, the HC (median score of 50 in both groups), followed by the Vitality domain (median score of 50; IQR, 45–70, for both groups); however, no improvement over time was observed and no statistically significant difference was observed between the two study groups (Table 6).

In the multivariate logistic regression model, some factors associated with poor PCS and MCS were found. The results indicated that having comorbidities (OR 2.22 95% CI 1.13–4.37 *p* = 0.013), sequelae attributed to COVID-19 (OR 5.93 95% CI 1.13–31.02 *p* = 0.000), persistence of COVID-19 symptoms (OR 2.83 95% CI 1.15–6.91 *p* = 0.000), fatigue (OR 2.18 95% CI 1.04–4.57 *p* = 0.038), and myalgia (OR 2.42 95% CI 1.00–5.87 *p* = 0.050) were risk factors for poor PCS. On the other hand, being female (OR 2.70 95% CI 1.41–5.15 *p* = 0.003), having anxiety (OR 5.54 95% CI 1.82–16.87 *p* = 0.003), and depression (OR 8.53 95% CI 1.08–67.39 *p* = 0.042) were associated with poor MCS (Table 7). 

## 4. Discussion

The main findings of our study were as follows: the proportion of outpatients with persistent COVID-19 symptoms was high (73.3%), similar to the proportion of hospitalized patients reported in the literature. The main persistent symptoms were fatigue and anxiety. No statistically significant difference in the SF-36 QoL scores and the frequency of persistent COVID-19 symptoms was found when comparing the ≤5 and >5 months groups, except for myalgia, which was less frequently observed in the >5 months group after COVID-19 infection. Female gender was associated with an increased risk of persistent symptoms. Having comorbidities and sequelae attributed to COVID-19, as well as persistent COVID-19 symptoms, were risk factors associated with poor PCS; whereas, female gender, anxiety, and depression were associated with poor MCS.

Regarding the general characteristics of the 206 outpatients who had recovered from COVID-19 included in our study, we found a higher proportion of females (62.6% females vs. 37.4% male), which corresponds with other studies performed on outpatients. For example, Logue et al., Anastasio. et al., and Nehme. et al. found a higher proportion of females, constituting 58%, 70.06%, and 63.8%, respectively [12,27,28]. Conversely, a higher proportion of males were reported when studies evaluated hospitalized patients; for example, Eberst et al. reported that most of the patients hospitalized in intensive care units were male, constituting 78.8% [29].

In our study of outpatients, the prevalence of comorbidities was lower than that reported in other studies with inpatients. Eberst et al. described that most ICU patients (92.9%) had at least one comorbidity [29]. Similarly, in their study on hospitalized patients, the PHSOP-COVID Collaborative Group described that 72.4% of patients had more than one comorbidity at 5 months of follow-up after hospitalization [15]. In our study, associated chronic diseases were present in 36.4%, with overweight being the most frequent (14.1%), followed by depression (9.2%), hypertension (7.3%), and diabetes (4.9%). Similarly, Logue et al. found a low rate of comorbidities in their study on COVID-19 outpatients, where only 27.6% had comorbidities, with hypertension (13%) and diabetes (5.1%) being the most frequent [12].

As mentioned before, the proportion of our study patients with at least one persistent COVID-19 symptom was high (73.3%) when compared with other studies. For example, Desgranges et al. and Logue et al. observed at least one persistent COVID-19 symptom in 53% and 32.7% of their respective studied outpatients [11,12]. Otherwise, when comparing our findings in outpatients with studies performed in hospitalized patients, the proportion of those with persistent symptoms is similar. In their systematic review and meta-analysis of hospitalized COVID-19 patients, López León et al. reported that 80% had at least one or more persistent symptom (the time for the different studies ranged from 14 to 110 days) [30]. Finding the cause for the different percentages observed among the studies is beyond the scope of the present investigation; however, the observed difference could be associated with several factors including ethnicity, gender, body mass index (BMI), and age [15,31].

In our study, the most frequent persistent COVID-19 symptom was fatigue (36.9%), which is consistent with the findings of Desgranges et al., as they observed fatigue as the main persistent COVID-19 symptom in 32% of ambulatory patients 3 months after SARS-CoV-2 infection [11]. Similarly, Logue et al. described fatigue as the most frequent symptom in 13.6%, although they included both ambulatory and hospitalized patients, most of whom were outpatients (150 outpatients vs. 16 inpatients) [12].

We observed that the frequency of persistent COVID-19 symptoms was similar in the ≤5 and >5 months groups, except for myalgia, which was less frequent >5 months after COVID-19 infection (26.2% vs. 14.1%, *p* ≤ 0.038). It is important to note that most symptoms are usually present for >5 months, such as fatigue, dyspnea, joint pain, cough, headache, anxiety, and depression. Other researchers have reported persistence of COVID-19 symptoms several months after SARS-CoV-2 infection; for example, in a cohort study of hospitalized patients by the PHSOP collaborative group, they observed that only a minority of the participants felt fully recovered 1 year after hospital discharge following SARS-CoV-2 infection. Interestingly, they found that 13 proteins (interleukin-6, erythropoietin, CD83, among others) were increased in participants in the severe physical and mental health impairment cluster compared with the mild physical and mental health impairment cluster of patients [15]. The cause of persistent COVID-19 symptoms remains unclear, but inflammatory modulators could play an important role; therefore, future studies focused on ameliorating the inflammatory profile in patients with long-COVID could be important to improve the well-being and QoL in this population [32]. In addition, we suggest a longer follow-up for ambulatory patients after COVID-19, as well as in hospitalized patients, because the percentage of those with persistent symptoms remains high even months after the infection.

The quality of life in COVID-19 outpatients is a topic that has been poorly evaluated. Logue et al. mention that a total of 29% of outpatients reported a worse QoL, but they do not explain which questionnaire they used to measure the QoL nor the degree of affection. In our survey, we used the SF-36 questionnaire to assess QoL and observed that the HC domain was the most affected score (median score 50, IQR 25–75) followed by the VT (median score 55, IQR 45–70) and the MH domain (median score 60, IQR 48–76). Temperoni et al. evaluated QoL in their study on COVID-19 middle-aged adults, and similarly reported that outpatients had lower scores in the VT domain (47.65 ± 21.22); however, they also observed lower scores in the RP (Role Physical 31.12 ± 42.86) and RE domains (45.57 ± 45.99) [33]. This is consistent with findings by Chen et al., who also found lower scores in the RP (72.29 ± 36.40) and RE domain (66.64 ± 45.62), although all of their patients were hospitalized [34]. In contrast, Zhou et al. evaluated the QoL with the SF-36 in non-severe but hospitalized patients; they found lower scores in the PF (Physical function domain) and GH (General health domain), with parameters below 60 [31]. These differences among the affected parameters in the SF-36 questionnaire with respect to QoL could be explained by the fact that most studies assessed quality of life in hospitalized patients with mild to severe disease; however, further studies will be needed to corroborate our findings. 

Regarding the PCS and MCS, we found that the oblique MCS (median score 45.4, IQR 38.7–52.1) presented lower scores than the oblique PCS (median scores 50.2 IQR 44.6–55.2). Similar to our study, both Temperoni et al. in their study with COVID-19 outpatients, and Chen et al. in COVID-19 hospitalized patients, observed lower scores in the MCS than in PCS after 1 month; however, they did not mention which method was used (orthogonal or oblique) to calculate these domains [33,34]. This trend was also observed in an Italian study by Anastasio et al., in which they found lower MCS scores than PCS scores in hospitalized patients who had mild to severe COVID-19; however, they used the SF-12 questionnaire to assess the QoL [27]. In contrast, in a study that we recently published, we found that the PCS score was lower than the MCS score in hospitalized patients 90 days after COVID-19 infection [35]. More studies are needed to confirm these results; however, we consider that hospitalized patients may have lower scores in the physical domains than ambulatory patients, which is probably related to the length of stay, more severe disease, and use of mechanical ventilation [36].

Quality of life and persistent symptoms could be affected by some risk factors. We observed that female gender was associated with an increased risk of persistent symptoms after SARS-CoV-2 infection (OR of 2.95, 95% CI 1.56–5.57). Accordingly, Jacobs et al. described that females were statistically more likely to have persistent symptoms with an OR of 2.16 (male vs. female OR 0.46, 95% CI 2.21–33.88 *p* = 0.035) [37]. In addition, the PHSOP-COVID Collaborative Group reported female gender as an independent factor associated with a lower probability of recovery from COVID-19 at 1 year (OR 0.68, 95% CI 0.46–0.99) [15]. This gender difference may be explained by the combination of biological sex and gender factors, such as behavioral/social roles [38]. Additionally, in their review on COVID-19 and gender differences, Haitao et al. mention that immunomodulation by sex hormones such as testosterone, estrogens, and X-linked gene expression are risk factors for outcome, prognosis, and mortality related to SARS-CoV-2 infection [38].

Intriguingly, we found that smoking was associated with a protective effect (OR 0.42, 95% CI 0.21–0.82, *p* = 0.012). Regarding this, in their review about nicotine and COVID-19, Tizabi. et al. mentioned substantial evidence for the adverse effects of smoking on the severity of progression and mortality associated with COVID-19; however, they support a potential therapeutic role for nicotine and nicotinic agonists in COVID-19, owing to their varied effects including mood regulation, anti-inflammatory, and purported interference with SARS-CoV-2 entry and/or replication [39]. It is important to emphasize that although we observed that smoking was associated with a protective effect against persistent COVID-19 symptoms, we do not encourage smoking because of the well-documented negative side effects [40,41].

Regarding risk factors associated with poor HRQoL (poor PCS and MCS), we found that having comorbidities and sequelae attributed to COVID-19, and the presence of persistent COVID-19 symptoms, mainly fatigue or myalgia, were associated with poor PCS, whereas risk factors for poor MCS were female, anxiety, and depression. Consistent with our findings, Qu et al. found that having physical symptoms after discharge (OR = 6.68, 95% CI 4.22–10.59) was associated with poor PCS [42]. Furthermore, Chen et al. observed that being female was associated with a poor MCS (OR = 2.22, 95% CI 1.30–3.81, *p* = 0.005) [34]. The reason why females have lower MCS scores than males is not known at this point; however, some studies have mentioned that this could be influenced by some biological, societal, and psychological factors that may determine the emotional state of females [21]. Regarding societal and psychological factors, studies have shown that the general population, who had experienced different levels of psychosocial stressors amid the COVID-19 pandemic, had developed mental health problems [43,44]. Individuals with a COVID-19 diagnosis had profound psychological distress, anxiety, depression, and other mental health problems compared to those who were not infected. Although the apparently high prevalence of such symptoms could be related to coronavirus infection, it is more probable that it is associated with the fear of adverse health outcomes due to COVID-19 and the mentioned societal and psychosocial stressors. The latter also highlights the mental health aspect of a physical health problem among those individuals [44]. In any case, this is an area of opportunity, an effort must be made to identify vulnerable individuals and grant them access to mental health services.

Our study has some limitations, such as the sample size, which could decrease the power of the study and may not reflect the entire population. In addition, we did not assess the presence of symptoms prior to COVID-19; ideally, persistent symptoms after COVID-19 should be evaluated before and after disease. Additionally, a long-term follow-up of outpatients would be desirable to observe how long symptoms continue and whether there is an improvement or worsening in quality of life because, so far, there is no established treatment for long-COVID-19. 

## 5. Conclusions

In conclusion, we found that most outpatients had persistent COVID-19 symptoms after infection, with the most common being fatigue, anxiety, and headache. No statistically significant difference in the SF-36 QoL scores and the frequency of persistent COVID-19 symptoms was found when comparing the ≤5 and >5 months groups, except for myalgia, which was less frequently observed in the >5 months group after COVID-19. In terms of quality of life (QoL), the most affected parameters were HC, VT, and MH. Being female and having depression or anxiety was associated with poor MCS; on the other hand, having sequelae, persistent symptoms, and fatigue or myalgia were associated with poor PCS. The proportion of persistent symptoms in ambulatory patients is high; therefore, we consider that as well as patients discharged from the hospital after SARS-CoV-2 infection, those under ambulatory management should be followed in a post-COVID-19 clinic and provided with rehabilitation and psychological therapy to improve their QoL, while finding a better way to treat “long- COVID”.

## Figures and Tables

**Table 1:** General characteristics of ambulatory patients recovered from COVID-19.

	Study Group, *n* = 206
Age, median (IQR)	28 (22–45.5)
Male, *n*(%)	77 (37.4%)
Female, *n*(%)	129 (62.6%)
Smoking, *n*(%)	52 (25.2%)
Comorbidities, *n*(%)	75 (36.4%)
Diabetes, *n*(%)	10 (4.9%)
Hypertension, *n*(%)	15 (7.3%)
Overweight, *n*(%)	29 (14.1%)
Cardiopathy, *n*(%)	3 (1.5%)
Dyslipidemia, *n*(%)	5 (2.4%)
Depression, *n*(%)	19 (9.2%)
Asthma, *n*(%)	9 (4.4%)
Autoimmune disease, *n*(%)	4 (1.9%)
Home supplemental oxygen, *n*(%)	23 (11.2%)
Complications/sequelae attributed to COVID-19, *n*(%)	16 (7.8%)
Inability to walk, *n*(%)	12 (5.8%)
Neuro/myopathy, *n*(%)	1 (0.5%)
Myocardial infarction/ heart failure, *n*(%)	1 (0.5%)
Treatments used during disease, *n*(%)	
Macrolide, *n*(%)	72 (35%)
Ivermectin, *n*(%)	47 (22.8%)
Vitamin D, *n*(%)	56 (27.2%)
Corticosteroid, *n*(%)	56 (27.2%)

**Table 2:** Influence of gender, comorbidities, and treatment on the persistence of symptoms in outpatients who had recovered from COVID-19.

	Study Group, *n* = 206			
	Persistent Symptoms (*n* = 151)	Asymptomatic (*n* = 55)	*p*-Value *	OR (95 % CI)
Female, *n*(%)	105 (69.5)	24 (43.6)	0.001	2.95 (1.56–5.57)
Age in years, median (IQR)	30 (22–49)	28 (23–46)	0.941	
Hypertension, *n*(%)	13 (8.6)	2 (3.6)	0.253	2.50 (0.54–11.4)
Smoking, *n*(%)	31 (20.5)	21 (38.2)	0.012	0.42 (0.21–0.82)
Overweight, *n*(%)	24 (15.9)	5 (9.1)	0.262	1.89 (0.68–5.23)
Diabetes, *n*(%)	7 (4.6)	3 (5.5)	1.000	0.84 (0.21–3.38)
Macrolides treatment	62 (41.1)	10 (18.2)	0.003	3.13 (1.47–6.67)
Vitamin D treatment	43 (28.5)	13 (23.6)	0.596	1.29 (0.63–2.63)
Corticosteroids	46 (30.5)	10 (18.2)	0.110	1.97 (0.91–4.25)

OR, odds ratio; * Statistical significance was calculated using the χ2 test for percentages, and Mann–Whitney U test for quantitative variables with non-parametric distribution.

**Table 3:** Comparison of general characteristics and comorbidities between groups.

Study Group, *n* = 206			
Variable	≤5 Months, (*n* = 107)	>5 Months, (*n* = 99)	*p*-Value *
Days after the onset of COVID-19 symptoms, median (IQR)	107 (85–130)	211 (176–269)	<0.001
Male, *n*(%)	36 (33.6)	41 (41.4)	0.31
Female, *n*(%)	71 (66.4)	58 (58.6)	
Smoking, *n*(%)	22 (20.6)	30 (30.3)	0.112
Comorbidities, *n*(%)	35 (32.7)	40 (40.4)	0.31
Diabetes, *n*(%)	7 (6.5)	3 (3)	0.335
Hypertension, *n*(%)	8 (7.5)	7 (7.1)	1.000
Dyslipidemia, *n*(%)	2 (1.9)	3 (3)	0.673
Overweight, *n*(%)	18 (16.8)	11 (11.1)	0.316
Depression, *n*(%)	9 (8.4)	10 (10.1)	0.811
Asthma, *n*(%)	5 (4.7)	4 (4)	1.000
Home supplemental oxygen, *n*(%)	12(11.2)	11 (11.1)	1.000

* Statistical significance was calculated using Mann–Whitney U test for quantitative variables with non-parametric distribution, and χ^2^ test for percentages.

**Table 4:** COVID-19 persistent symptoms at different times after infection: a comparison between groups.

Study Group, *n* = 206			
Variable	≤5 Months, (*n* = 107)	>5 Months, (*n* = 99)	*p*-Value *
Persistence of any COVID-19 symptoms, *n*(%)	78 (25.7)	73 (21.8)	0.510
Fatigue, *n*(%)	40 (37.4)	36 (36.4)	0.886
Dyspnea, *n*(%)	23 (21.5)	19 (19.2)	0.731
Joint pain, *n*(%)	16 (15)	16 (16.2)	0.849
Chest pain, *n*(%)	12 (11.2)	6 (6.1)	0.223
Cough, *n*(%)	9 (8.4)	11 (11.1)	0.639
Smell dysfunction, *n*(%)	20 (18.7)	25 (25.3)	0.312
Headache, *n*(%)	24 (22.4)	27 (27.3)	0.518
Sicca syndrome, *n*(%)	15 (14)	17 (17.2)	0.568
Red eyes, *n*(%)	6 (5.6)	4 (4)	0.750
Sputum production, *n*(%)	9 (8.4)	7 (7.1)	0.798
Lack of appetite, *n*(%)	14 (13.1)	8 (8.1)	0.268
Sore throat, *n*(%)	6 (5.6)	9 (9.1)	0.424
Vertigo, *n*(%)	4 (3.7)	6 (6.1)	0.526
Myalgia, *n*(%)	28 (26.2)	14 (14.1)	0.038
Diarrhea, *n*(%)	4 (3.7)	4 (4%)	1.000
Anxiety, *n*(%)	26 (24.3)	28 (28.3)	0.530
Depression, *n*(%)	13 (12.1)	19 (19.2)	0.182
Alopecia, *n*(%)	28 (26.2)	19 (19.2)	0.249

* Statistical significance was calculated using χ^2^ test.

**Table 5:** Quality of life (SF-36 scores) in outpatients who had recovered from COVID-19.

Study Group, *n* = 206	
SF-36 Scale	Score, Median (IQR)
Physical function	95 (80–100)
Role physical function	100 (50–100)
Body pain	90 (68–100)
General health	65 (50–80)
Mental health	60 (48–76)
Role emotional	100 (33–100)
Vitality	55 (45–70)
Social function	75 (50–100)
Health change	50 (25–75)
Correlated physical component summary	50.2 (44.6–55.2)
Correlated mental component summary	45.4 (38.7–52.1)

**Table 6:** SF-36 scores of outpatients who had recovered from COVID-19 at different times: a comparison between groups.

Study Group, *n* = 206			
	≤5 Months (*n* = 107)	>5 Months (*n* = 99)	*p*-Value *
	Score, Median (IQR)	Score, Median (IQR)	
PF	95 (80–100)	86.92 (80–100)	0.478
RP	100 (50–100)	100 (75–100)	0.299
RE	65.14 (33–100)	100 (33–100)	0.412
VT	55 (45–70)	55 (45–70)	0.477
MH	64 (50–76)	60 (48–76)	0.783
SF	75 (63–88)	75 (50–100)	0.784
BP	90 (68–100)	90 (68–100)	0.473
GH	65 (50–80)	60 (50–75)	0.730
HC	50 (25–50)	50 (25–75)	0.204
PCSc	50.74 (40.80–55.29)	50.13 (44.28–55.41)	0.823
MCSc	45.13 (39.09–52.16)	45.69 (38.50–52.16)	0.900

PF: Physical function, RP: Role physical function, BP: body pain, GH: general health, VT: vitality, SF: social function, RE: Role emotional, MH: mental health, HC: health change, PCSc: Correlated physical component summary, MCSc: Correlated mental component summary. * Statistical significance was calculated using Mann–Whitney U test.

**Table 7:** Results of multivariate logistic regression for HQRL of COVID-19 outpatients.

Outcomes	Variables	Odds Ratio (95 % CI)	*p*-Value
Poor PCS	Comorbidities	2.22 (1.13–4.37)	0.021
	Sequalae	5.93 (1.13–31.02)	0.035
	Persistent symptoms	2.83 (1.15–6.91)	0.023
	Fatigue	2.18 (1.04–4.57)	0.038
	Myalgia	2.42 (1.00–5.87)	0.050
Poor MCS	Female	2.70 (1.41–5.15)	0.003
	Anxiety	5.54 (1.82–16.87)	0.003
	Depression	8.53 (1.08–67.39)	0.042

PCSc: Correlated physical component summary, MCSc: Correlated mental component summary.

## Data Availability

All relevant data are within the paper.

## References

[1] Lindert J., Jakubauskiene M., Bilsen J. (2021). The COVID-19 disaster and mental health-assessing, responding and recovering. Eur. J. Public Health.

[2] WHO (2022). Weekly Epidemiological Update on COVID-19—6 July 2022. https://www.who.int/publications/m/item/weekly-epidemiological-update-on-covid-19---6-july-2022.

[3] Merad M., Martin J.C. (2020). Author correction: Pathological inflammation in patients with COVID-19: A key role for monocytes and macrophages. Nat. Rev. Immunol..

[4] Jain U. (2020). Effect of COVID-19 on the Organs. Cureus.

[5] Carfì A., Bernabei R., Landi F. (2020). Persistent symptoms in patients after acute COVID-19. JAMA.

[6] Raveendran A.V., Jayadevan R., Sashidharan S. (2021). Long COVID: An overview. Diabetes Metab. Syndr..

[7] Huang C., Huang L., Wang Y., Li X., Ren L., Gu X., Kang L., Guo L., Liu M., Zhou X. (2021). 6-month consequences of COVID-19 in patients discharged from hospital: A cohort study. Lancet.

[8] Chopra V., Flanders S.A., O’Malley M., Malani A.N., Prescott H.C. (2021). Sixty-day outcomes among patients hospitalized with COVID-19. Ann. Intern. Med..

[9] Halpin S.J., McIvor C., Whyatt G., Adams A., Harvey O., McLean L., Walshaw C., Kemp S., Corrado J., Singh R. (2021). Postdischarge symptoms and rehabilitation needs in survivors of COVID-19 infection: A cross-sectional evaluation. J. Med. Virol..

[10] Evans R.A., McAuley H., Harrison E.M., Shikotra A., Singapuri A., Sereno M., Elneima O., Docherty A.B., Lone N.I., Leavy O.C. (2021). Physical, cognitive, and mental health impacts of COVID-19 after hospitalisation (PHOSP-COVID): A UK multicentre, prospective cohort study. Lancet Respir. Med..

[11] Desgranges F., Tadini E., Munting A., Regina J., Filippidis P., Viala B., Karachalias E., Suttels V., Haefliger D., Kampouri E. (2022). Post-COVID-19 syndrome in outpatients: A cohort study. J. Gen. Intern. Med..

[12] Logue J.K., Franko N.M., McCulloch D.J., McDonald D., Magedson A., Wolf C.R., Chu H.Y. (2021). Sequelae in adults at 6 months after COVID-19 infection. JAMA Netw. Open.

[13] Barker-Davies R.M., O’Sullivan O., Senaratne K.P.P., Baker P., Cranley M., Dharm-Datta S., Ellis H., Goodall D., Gough M., Lewis S. (2020). The stanford hall consensus statement for post-COVID-19 rehabilitation. Br. J. Sports Med..

[14] Von Elm E., Altman D.G., Egger M., Pocock S.J., Gøtzsche P.C., Vandenbroucke J.P. (2007). The strengthening the reporting of observational studies in epidemiology (STROBE) statement: Guidelines for reporting observational studies. Epidemiology.

[15] Evans R.A., Leavy O.C., Richardson M., Elneima O., McCauley H.J.C., Shikotra A., Singapuri A., Sereno M., Saunders R.M., Harris V.C. (2022). Clinical characteristics with inflammation profiling of long COVID and association with 1-year recovery following hospitalisation in the UK: A prospective observational study. Lancet Respir. Med..

[16] Levis B., Benedetti A., Thombs B.D., DEPRESsion Screening Data (DEPRESSD) Collaboration (2019). Accuracy of patient health questionnaire-9 (PHQ-9) for screening to detect major depression: Individual participant data meta-analysis. BMJ.

[17] Plummer F., Manea L., Trepel D., McMillan D. (2016). Screening for anxiety disorders with the GAD-7 and GAD-2: A systematic review and diagnostic metaanalysis. Gen. Hosp. Psychiatry.

[18] Vista de Evaluación del Estado de Salud con la Encuesta SF-36: Resultados Preliminares en México|Salud Pública de México. https://www.saludpublica.mx/index.php/spm/article/view/6138/7233.

[19] The MOS 36-Item Short-Form Health Survey (SF-36). I. Conceptual Framework and Item Selection—PubMed. https://pubmed.ncbi.nlm.nih.gov/1593914/.

[20] Barile J.P., Horner-Johnson W., Krahn G., Zack M., Miranda D., DeMichele K., Ford D., Thompson W.W. (2016). Measurement characteristics for two health-related quality of life measures in older adults: The SF-36 and the CDC healthy days items. Disabil. Health J..

[21] Del Core M.A., Ahn J., Wukich D.K., Liu G.T., Lalli T., VanPelt M.D., Raspovic K.M. (2018). Gender differences on SF-36 patient-reported outcomes of diabetic foot disease. Int. J. Low Extrem. Wounds.

[22] Laucis N.C., Hays R.D., Bhattacharyya T. (2015). Scoring the SF-36 in orthopaedics: A brief guide. J. Bone Joint Surg. Am..

[23] Ware J. (1994). SF-36 Physical and Mental Health Summary Scales: A User’s Manual.

[24] VassarStats. http://vassarstats.net/.

[25] Farivar S.S., Cunningham W.E., Hays R.D. (2007). Correlated physical and mental health summary scores for the SF-36 and SF-12 Health Survey, V.I. Health Qual. Life Outcomes.

[26] Hays R.D., Morales L.S. (2001). The RAND-36 measure of health-related quality of life. Ann. Med..

[27] Anastasio F., Barbuto S., Scarnecchia E., Cosma P., Fugagnoli A., Rossi G., Parravicini M., Parravicini P. (2021). Medium-term impact of COVID-19 on pulmonary function, functional capacity and quality of life. Eur. Respir. J..

[28] Nehme M., Braillard O., Chappuis F., Courvoisier D.S., Kaiser L., Soccal P.M., Reny J.L., Assal F., Bondolfi G., Tardin A. (2022). One-year persistent symptoms and functional impairment in SARS-CoV-2 positive and negative individuals. J. Intern. Med..

[29] Eberst G., Claudé F., Laurent L., Meurisse A., Roux-Claudé P., Barnig C., Vernerey D., Paget-Bailly S., Bouiller K., Chirouze C. (2022). Result of one-year, prospective follow-up of intensive care unit survivors after SARS-CoV-2 pneumonia. Ann. Intensive Care.

[30] Chen K.Y., Li T., Gong F.H., Zhang J.S., Li X.K. (2020). Predictors of health-related quality of life and influencing factors for COVID-19 patients, a follow-up at one month. Front. Psychiatry.

[31] Qu G., Zhen Q., Wang W., Fan S., Wu Q., Zhang C., Li B., Liu G., Yu Y., Li Y. (2021). Health-related quality of life of COVID-19 patients after discharge: A multicenter follow-up study. J. Clin. Nurs..

[32] Lopez-Leon S., Wegman-Ostrosky T., Perelman C., Sepulveda R., Rebolledo P.A., Cuapio A., Villapol S. (2021). More than 50 long-term effects of COVID-19: A systematic review and meta-analysis. Sci. Rep..

[33] Zhou F., Tao M., Shang L., Liu Y., Pan G., Jin Y., Wang L., Hu S., Li J., Zhang M. (2021). Assessment of sequelae of COVID-19 nearly 1 year after diagnosis. Front. Med..

[34] Jacobs L.G., Paleoudis E.G., Bari D.L.-D., Nyirenda T., Friedman T., Gupta A., Rasouli L., Zetkulic M., Balani B., Ogedegbe C. (2020). Persistence of symptoms and quality of life at 35 days after hospitalization for COVID-19 infection. PLoS ONE.

[35] Kim Y., Kim S.-W., Chang H.-H., Kwon K.T., Hwang S., Bae S. (2022). One year follow-up of COVID-19 related symptoms and patient quality of life: A prospective cohort study. Yonsei Med. J..

[36] Temperoni C., Grieco S., Pasquini Z., Canovari B., Polenta A., Gnudi U., Montalti R., Barchiesi F. (2021). Clinical characteristics, management and health related quality of life in young to middle age adults with COVID-19. BMC Infect. Dis..

[37] Muñoz-Corona C., Gutiérrez-Canales L.G., Ortiz-Ledesma C., Martínez-Navarro L.J., Macías A.E., Scavo-Montes D.A., Guaní-Guerra E. (2022). Quality of life and persistence of COVID-19 symptoms 90 days after hospital discharge. J. Int. Med. Res..

[38] Monti G., Leggieri C., Fominskiy E., Scandroglio A.M., Colombo S., Tozzi M., Moizo E., Mucci M., Crivellari M., Pieri M. (2021). Two-months quality of life of COVID-19 invasively ventilated survivors; an Italian single-center study. Acta Anaesthesiol. Scand..

[39] Haitao T., Vermunt J.V., Abeykoon J., Ghamrawi R., Gunaratne M., Jayachandran M., Narang K., Parashuram S., Suvakov S., Garovic V.D. (2020). COVID-19 and sex differences: Mechanisms and biomarkers. Mayo Clin. Proc..

[40] Tizabi Y., Getachew B., Copeland R.L., Aschner M. (2020). Nicotine and the nicotinic cholinergic system in COVID-19. FEBS J..

[41] Gandini S., Botteri E., Iodice S., Boniol M., Lowenfels A.B., Maisonneuve P., Boyle P. (2008). Tobacco smoking and cancer: A meta-analysis. Int. J. Cancer.

[42] Poudel R., Daniels L.B., DeFilippis A.P., Hamburg N.M., Khan Y., Keith R.J., Kumar R.S., Strokes A.C., Robertson R.M., Bhatnagar A. (2022). Smoking is associated with increased risk of cardiovascular events, disease severity, and mortality among patients hospitalized for SARS-CoV-2 infections. PLoS ONE.

[43] Roy D., Tripathy S., Kar S.K., Sharma N., Verma S.K., Kaushal V. (2020). Study of knowledge, attitude, anxiety & perceived mental healthcare need in Indian population during COVID-19 pandemic. Asian J. Psychiatry.

[44] Hossain M.M., Tasnim S., Sultana A., Faizah F., Mazumder H., Zou L., McKyer E.L.J., Ahmed H.U., Ma P. (2020). Epidemiology of mental health problems in COVID-19: A review. F1000Research.

